# Autophagy under glucose starvation enhances protein translation initiation in response to re‐addition of glucose in C2C12 myotubes

**DOI:** 10.1002/2211-5463.12970

**Published:** 2020-09-20

**Authors:** Naoya Nakai, Saki Kitai, Noriko Iida, Sachika Inoue, Kazuhiko Higashida

**Affiliations:** ^1^ Laboratory of Exercise Nutrition Department of Nutrition University of Shiga Prefecture Hikone Japan

**Keywords:** autophagy, glucose starvation, mTORC1, p70 S6 kinase, protein synthesis

## Abstract

Proteolysis is known to play a crucial role in maintaining skeletal muscle mass and function. Autophagy is a conserved intracellular process for the bulk degradation of proteins in lysosomes. Although nutrient starvation is known to induce autophagy, the effect of nutrient repletion following starvation on the mTOR pathway‐mediated protein translation remains unclear. In the present study, we examined the effect of glucose starvation on the initiation of protein translation in response to glucose re‐addition in C2C12 myotubes. Glucose starvation decreased the phosphorylation of p70 S6 kinase (p70S6K), a bonafide marker for protein translation initiation. Following re‐addition of glucose, phosphorylation of p70S6K markedly increased only in glucose‐starved cells. Inhibiting autophagy using pharmacological inhibitors diminished the effect of glucose re‐addition on the phosphorylation of p70S6K, whereas inhibition of the ubiquitin‐proteasome system did not exert any effect. In conclusion, autophagy under glucose starvation partially accounts for the activation of translation initiation by re‐addition of glucose.

## Graphical abstract

Cells starved of glucose for 24 h showed enhanced phosphorylation of p70S6K in response to re‐addition of glucose. Pharmacological inhibition studies revealed that autophagy under glucose starvation partially accounts for this effect. The results of the present study suggest that appropriate nutrient starvation can modulate cellular protein turnover and may be effective at improving muscle protein synthesis.

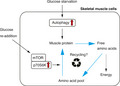

**Abbreviations**
2‐DG: 2‐deoxyglucose
Baf: bafilomycin
DMEM: Dulbecco's modified Eagle's medium
HG: high glucose
LC3: light chain 3
mTOR: mammalian target of rapamycin
NG: no glucose
p70S6K: p70 S6 kinase

The regulation of skeletal muscle mass depends on the balance between overall rates of protein synthesis and breakdown. The most effective way to enhance skeletal muscle is increasing protein synthesis and decreasing protein breakdown. However, growing evidence suggests that muscle protein breakdown is also essential for muscle hypertrophy.

The skeletal muscle proteolysis is regulated by two major arms: the autophagy‐lysosome pathway [1, 2] and the ubiquitin‐proteasome pathway [3, 4, 5]. Autophagy is a conserved intracellular process for bulk degradation of proteins and organelles in lysosomes [6]. In mammalian cells, the main regulators of autophagy are hormonal and nutrient factors [7, 8]. Glucose [9, 10, 11] and amino acid [8, 12, 13] deprivation/starvation is known to induce autophagy. However, the effects of glucose starvation on autophagy are comparatively less studied than those of amino acids starvation.

Proteolysis is known to play a crucial role in maintaining skeletal muscle mass and function. It has been reported that knock down of Atg7, a crucial autophagy gene, results in profound muscle atrophy [14]. Tribbles homologue 3 (TRB3) knockout in mice attenuated muscle fiber atrophy by increasing autophagy [15]. Overexpression of Sestrins, a family of stress‐inducible metabolic regulators, prevents aging‐ and disuse‐induced muscle atrophy by upregulating autophagy [16]. The functional overload‐induced muscle hypertrophy is associated with an increase in ubiquitin‐proteasome activity [17]. Proteasome dysfunction in Rpt3‐deficient satellite cells impairs proliferation and differentiation capacity, resulting in defective muscle regeneration [18].

The serine/threonine protein kinase mammalian target of rapamycin (mTOR) is one of the critical factors regulating overall protein synthesis and breakdown. There are two functionally distinct mTOR complexes, mTORC1 and mTORC2 [19]. mTORC1 enhances protein translation through phosphorylation of p70 S6 kinase (p70S6K) and 4E‐binding protein‐1 (4E‐BP1) in response to hormonal and/or nutritional stimuli [20]. We have previously reported that insulin, mechanical stress, and branched‐chain amino acids like leucine can activate the p70S6K in C2C12 myotubes [21, 22]. Under nutrient‐rich conditions, mTORC1 stimulates cellular growth and suppresses autophagy, whereas inhibition of mTORC1 activity has been shown to increase autophagy [23].

In the present study, we examined the effect of glucose starvation on protein translation initiation in response to re‐addition of glucose in C2C12 myotubes. Our findings indicate that 24‐h glucose starvation decreased the signals for protein translation initiation. However, only in glucose‐starved cells showed enhanced phosphorylation of p70S6K in response to re‐addition of glucose. Pharmacological inhibition studies revealed that autophagy under glucose starvation partially accounts for this effect. The results of the present study suggest that appropriate nutrient starvation can modulate the cellular protein turnover, and may be effective at improving muscle protein synthesis.

## Materials and methods

### Materials

Dulbecco's modified Eagle's medium (DMEM, catalogue number D5796), fetal bovine serum (FBS, F2442), and Penicillin‐Streptomycin Solution (P7539) were purchased from Sigma‐Aldrich (St. Louis, MO, USA). DMEM without glucose (A1443001) was purchased from Thermo Fisher Scientific (Rockford, IL, USA). Antibodies against total‐p70S6K (9202), phospho‐T389‐p70S6K (9205), autophagy marker light chain 3 (LC3, 4599), and β‐actin (4970) were obtained from Cell Signaling Technology (Beverly, MA, USA). Antibodies against multi‐ubiquitinated protein (D058‐3) were obtained from MBL (Nagoya, Japan). Bafilomycin A1 (Baf, ab120497) was obtained from Abcam (Cambridge, UK). MG132 (M7449) was obtained from Sigma‐Aldrich.

### C2C12 cell culture

C2C12 cells (a mouse myoblast cell line) were cultured in DMEM, supplemented with 25 mm glucose, 10% FBS, 100 units·mL^−1^ penicillin, and 100 mg·mL^−1^ streptomycin sulfate in a 5% CO_2_‐humidified chamber at 37 °C. Cells were grown to approximately 90% confluence, and then, the culture medium was then replaced with differentiation medium (DMEM supplemented with 25 mm glucose, 2% horse serum, 100 units·mL^−1^ penicillin, and 100 mg·mL^−1^ streptomycin sulfate). Differentiation was carried out for 4 days to form multi‐nucleated myotubes. Differentiation medium was changed every 48 h.

### Glucose starvation and re‐addition

Differentiated C2C12 cells were divided into two groups that were cultured for additional 24 h with differentiation medium supplemented with or without glucose (high glucose, 25 mm: HG; no glucose: NG). Then, glucose was added to half of each group. After 30 min of incubation, all cells were rinsed with ice‐cold PBS and lysed with RIPA lysis buffer (Santa Cruz Biotechnology, Dallas, TX, USA). In experiments with pharmacological inhibitors, cells were incubated with Bafilomycin (at a final concentration of 20 nm) or MG132 (20 μm) for 24 h.

### Western blotting

Protein concentration in the cell lysate was determined using a BCA protein assay kit (Thermo Fisher Scientific). Equal amounts of total cellular protein were subjected to SDS/PAGE and transferred to polyvinylidene difluoride membranes (Immobilon‐P; Merck Millipore, Burlington, MA, USA) for immunoblotting. The membrane was blocked with Tris‐buffered saline (pH 7.4), containing 0.05% (v/v) Tween‐20 and 5% skimmed milk. Following an overnight incubation with the indicated primary antibodies, the membranes were washed and then incubated with horseradish peroxidase‐conjugated anti‐rabbit IgG or anti‐mouse IgG for 1 h at room temperature. Immuno‐reactive protein bands were visualized by Immobilon ECL Ultra Western HRP Substrate (Thermo Fisher Scientific) with an ImageQuant LAS500 image analyzer (GE Healthcare UK Limited, Chicago, IL, USA). The band intensity was quantified using a computer analysis package (imagej, open sourse software).

### Intracellular ATP assays

Intracellular ATP concentration was measured using an intracellular ATP determination kit (Toyo B‐net, Tokyo, Japan) following the manufacturer's instructions. For this assay, C2C12 myotubes were cultured in 96‐well plates.

### Statistical analyses

Data are presented as mean, and error bars represent SD. Statistical analysis for multiple comparisons was performed using one‐way or two‐way ANOVA followed by the Scheffe's *post hoc* test. Differences were considered statistically significant at *P* < 0.05.

## Results

### Effect of glucose starvation and re‐addition on the phosphorylation of p70S6K

**Fig. 1:**
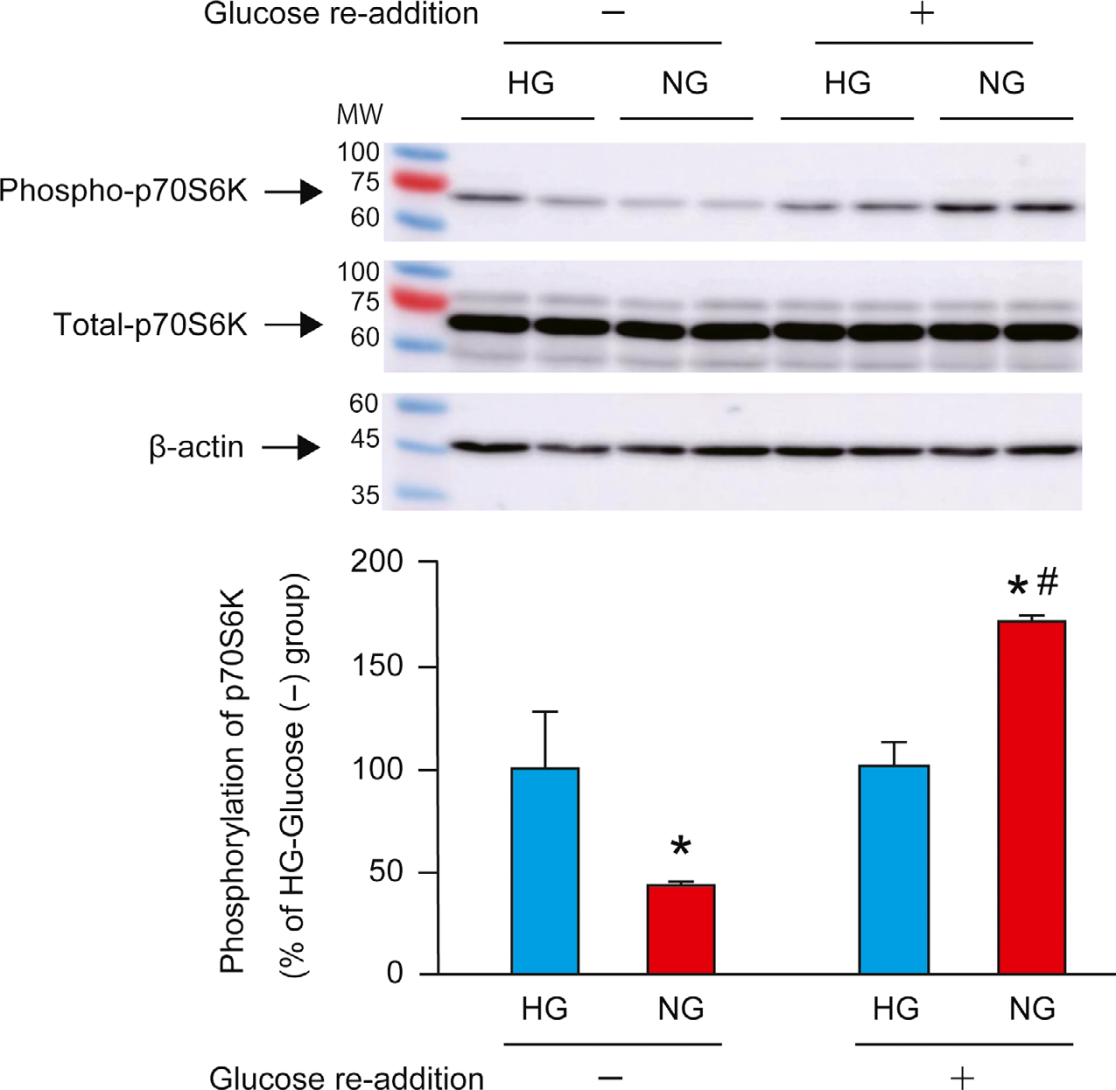
Effect of glucose starvation and re‐addition on p70S6K phosphorylation. C2C12 myotubes were cultured in HG or NG medium for 24 h, and half of each group was subjected to glucose re‐addition for 30 min. A representative western blot image for phospho‐p70S6K, total‐p70S6K, and β‐actin is shown in the upper panel. Quantitative data for phosphorylation of p70S6K are shown in the lower panel. Data are expressed as percentages relative to the HG‐Glucose (−) group (100%). The difference was analyzed using two‐way ANOVA followed by the Scheffe's *post hoc* test. Data are presented as mean, and error bars represent SD (*n* = 6). *P < 0.05 vs. HG group. ^#^ *P* < 0.05 vs. Glucose (−) group. MW, molecular weight marker.

The phosphorylation‐induced activation of the mTORC1/p70S6K pathway is a key step in regulation of translation initiation. The phosphorylation of p70S6K at threonine 389 (T389) is positively correlated with its kinase activity [24] and is associated with the increased activation of protein translation initiation [25]. Glucose starvation for 24 h significantly decreased the phosphorylation of p70S6K (Fig. 1). Addition of 5.5 mm glucose significantly increased the phosphorylation of p70S6K in the NG group compared with that in the HG group. However, total protein levels of p70S6K were not affected by glucose starvation and/or glucose re‐addition.

### Effect of glucose concentration on the phosphorylation of p70S6K after glucose starvation

**Fig. 2:**
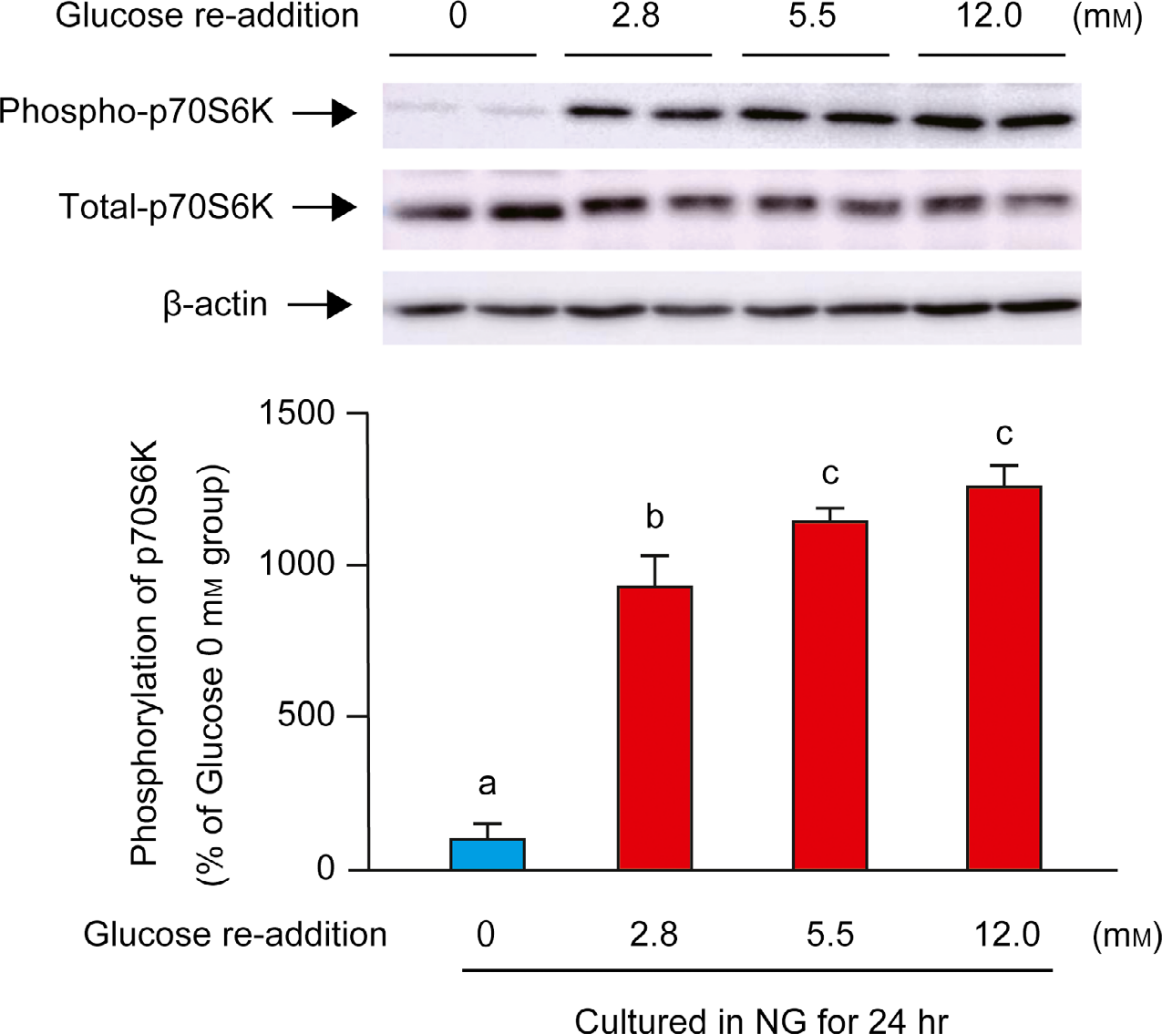
Effect of glucose concentration on the phosphorylation of p70S6K following glucose starvation. C2C12 myotubes were cultured in NG medium for 24 h, and different amounts (final concentration at 0, 2.8, 5.5, and 12.0 mm) of glucose were added to the cells and incubated for 30 min. A representative western blot image for phospho‐p70S6K, total‐p70S6K, and β‐actin is shown in the upper panel. Quantitative data for phosphorylation of p70S6K are shown in the lower panel. Data are represented as percentages relative to Glucose 0 mm group (100%). The difference was analyzed using one‐way ANOVA followed by the Scheffe's *post hoc* test. Data are presented as mean, and error bars represent SD (*n* = 4). Values with different letters are significantly different (*P* < 0.05).

The phosphorylation status of p70S6K was determined in response to varying amounts of glucose (final concentrations of 2.8, 5.5, and 12.0 mm) following glucose starvation for 24 h. After 30 min of incubation with increasing concentrations of glucose, the phosphorylation of p70S6K was significantly higher than in the 0 mm group in all cases (Fig. 2). The phosphorylation of p70S6K in 5.5 and 12.0 mm groups was higher than that in the 2.8 mm group, whereas the 5.5 and 12.0 mm groups showed the same level.

### Effect of glucose starvation and re‐addition on the concentration of intracellular ATP

**Fig. 3:**
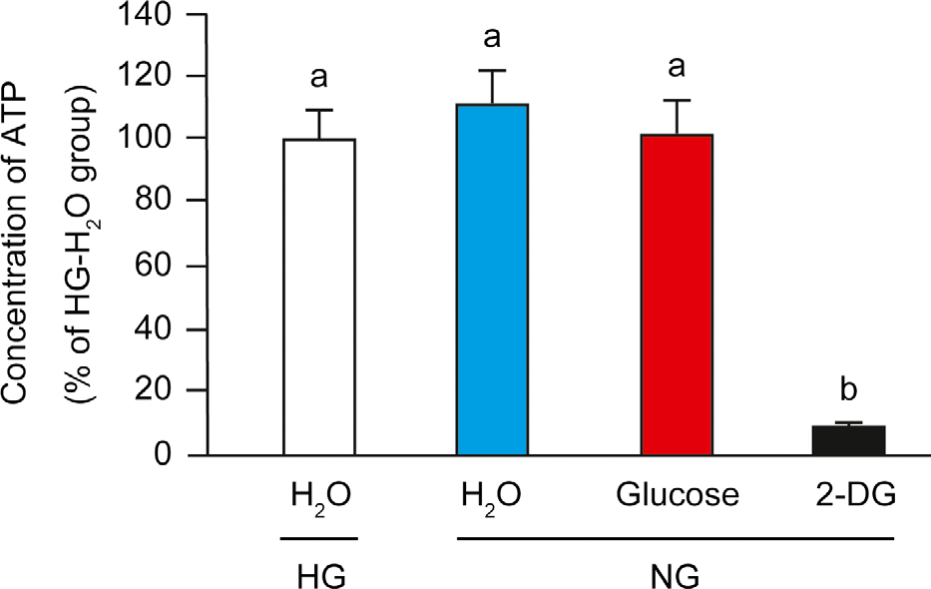
Effect of glucose starvation and re‐addition on intracellular ATP. C2C12 myotubes were cultured in HG or NG medium for 24 h. Cells were lysed at 30 min after the addition of H_2_O or glucose or 2‐DG. Data are expressed as percentages relative to the HG‐H_2_O group (100%). The difference was analyzed using one‐way ANOVA followed by the Scheffe's *post hoc* test. Data are presented as mean, and error bars represent SD (*n* = 5). Values with different letters are significantly different (*P* < 0.05).

Next, we found the concentration of intracellular ATP was not affected either by glucose starvation or glucose re‐addition in our system (Fig. 3). As a positive control, addition of 2‐deoxyglucose (2‐DG), a nonmetabolizable glucose analog that inhibits the glycolytic pathway, markedly decreased the concentration of intracellular ATP.

### Effect of proteasome inhibitor on glucose re‐addition

**Fig. 4:**
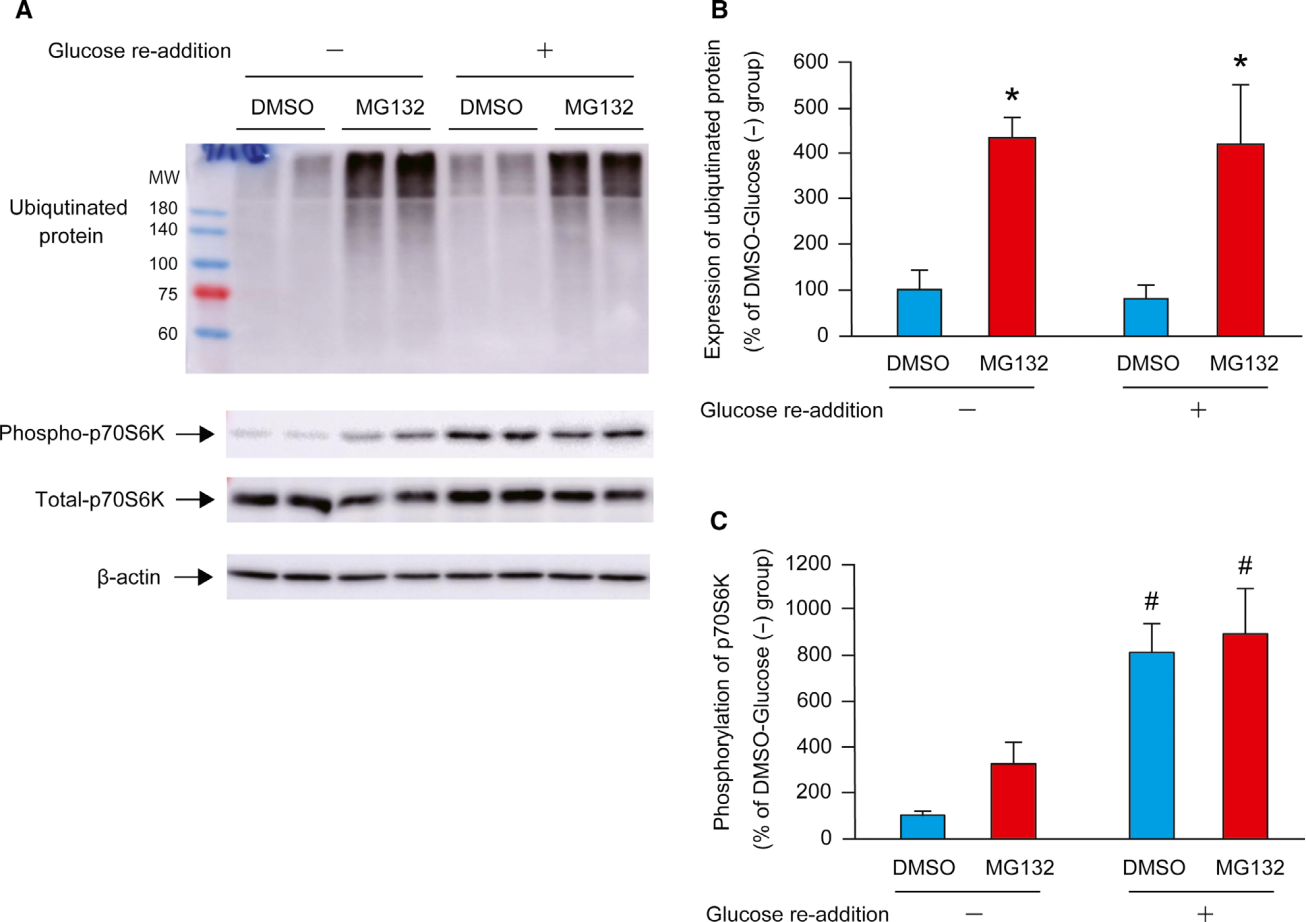
Effect of proteasome inhibitor on glucose re‐addition. C2C12 myotubes were cultured in NG medium for 24 h with or without MG132, dissolved in DMSO. Equal volume of DMSO was added to the control cells. Cells were lysed at 30 min after the re‐addition of 5.5 mm glucose. A representative western blot image for ubiquitinated protein, phospho‐p70S6K, total‐p70S6K, and β‐actin is shown in the left panel (A). Quantitative data for ubiquitinated protein are shown and expressed as percentages relative to the DMSO‐Glucose (−) group (100%) (B). Quantitative data for phosphorylation of p70S6K are shown and expressed as percentages relative to the DMSO‐Glucose (−) group (100%) (C). The difference was analyzed using two‐way ANOVA followed by the Scheffe's *post hoc* test. Data are presented as mean, and error bars represent SD (*n* = 4). *P < 0.05 vs. DMSO group. ^#^ *P* < 0.05 vs. Glucose (−) group. MW, molecular weight marker.

To determine the effect of proteasome inhibitor on glucose re‐addition, cells were treated with 20 μm MG132 (dissolved in DMSO) or vehicle. MG132 is a potent cell‐permeable proteasome inhibitor that reduces the degradation of ubiquitinated proteins in mammalian cells. The abundance of ubiquitinated protein was significantly higher in the MG132 groups than in the vehicle (Fig. 4A,B). The phosphorylation of p70S6K in response to glucose re‐addition was comparable between DMSO and MG132 groups (Fig. 4A,C).

### Effect of autophagy inhibitor on glucose re‐addition

**Fig. 5:**
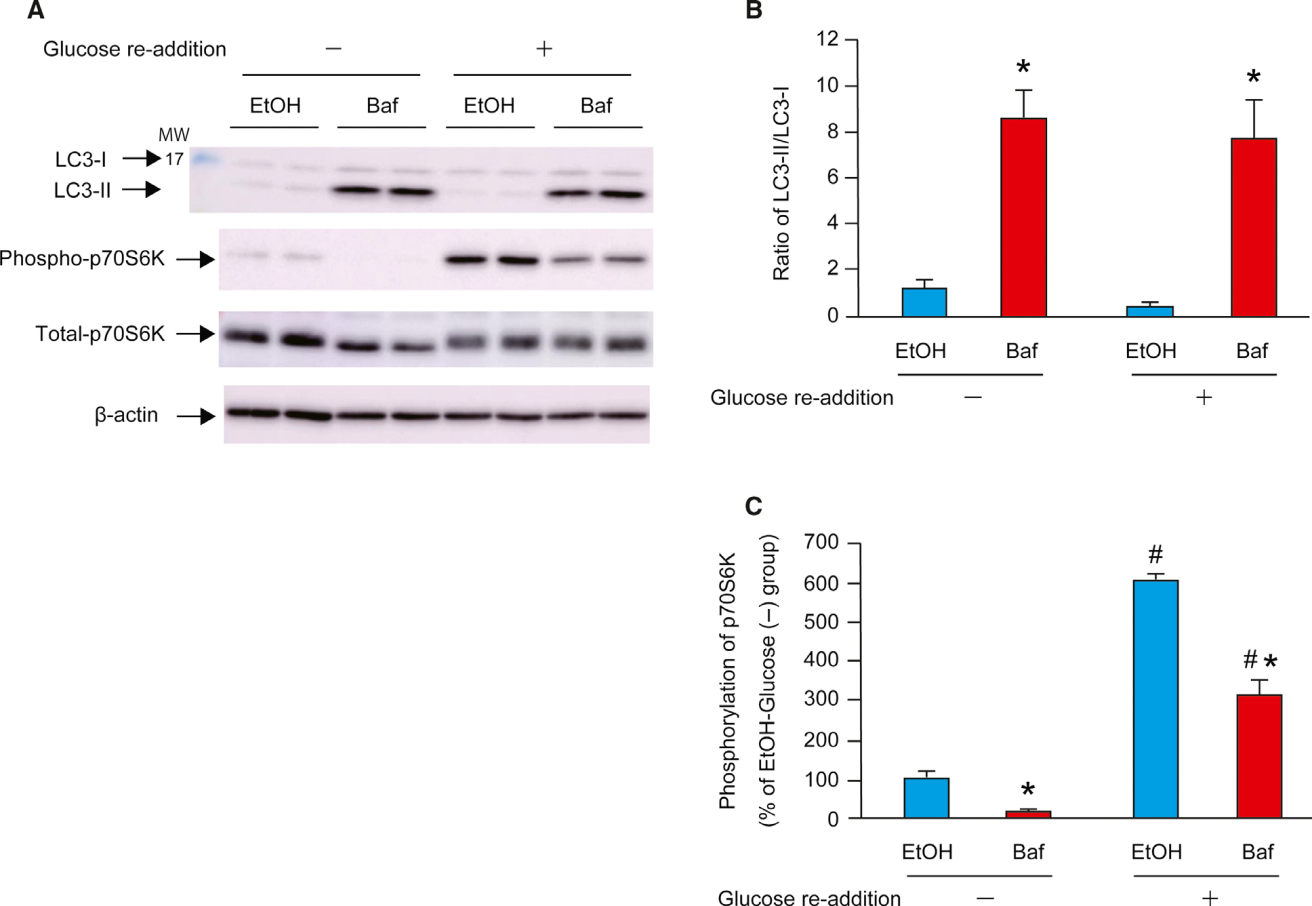
Effect of autophagy inhibitor on glucose re‐addition. C2C12 myotubes were cultured in NG medium for 24 h with or without bafilomycin (Baf), dissolved in EtOH. Equal volume of EtOH was added to the control cells. Cells were lysed at 30 min after the re‐addition of 5.5 mm glucose. A representative western blot image for LC3‐I, LC3‐II, phospho‐p70S6K, total‐p70S6K, and β‐actin is shown in the left panel (A). Quantitative data for ratio of LC3‐II/LC3‐I are shown in the panel (B). Quantitative data for phosphorylation of p70S6K are shown in the panel (C). Data are expressed as percentages relative to the EtOH‐Glucose (−) group (100%). The difference was analyzed using two‐way ANOVA followed by the Scheffe's *post hoc* test. Data are presented as mean, and error bars represent SD (*n* = 4). *P < 0.05 vs. EtOH group. ^#^ *P* < 0.05 vs. Glucose (−) group. MW, molecular weight marker.

To determine the effect of autophagy inhibitor on glucose restoration, 20 nm bafilomycin (Baf, dissolved in ethanol) was added to the cells. LC3 is a mammalian ortholog of yeast ATG8. The conversion of LC3‐I to LC3‐II serves as a specific marker for autophagy in mammalian cells [26, 27]. The LC3‐II/LC3‐I ratio was markedly higher in the Baf group than in the EtOH group after 24 h of glucose starvation (Fig. 5A,B). The phosphorylation of p70S6K in the glucose (−) group was significantly decreased by addition of Baf (Fig. 5A,C). The phosphorylation of p70S6K in response to glucose re‐addition was partly inhibited by Baf.

## Discussion

In the present study, we examined the effects of glucose starvation on protein translation initiation in response to glucose re‐addition in C2C12 myotubes. The results suggest that autophagy under glucose starvation partially accounts for the activation of translation initiation by glucose re‐addition.

Glucose starvation for 24 h decreases the phosphorylation levels of p70S6K. This is in line with previous studies suggesting that glucose is essential to maintain the phosphorylation levels of p70S6K [10, 11]. In addition, we found that re‐addition of glucose augmented the phosphorylation levels of p70S6K in 24‐h glucose‐starved cells but not in the nonstarved myotubes. These results suggest that 24‐h glucose starvation modulates cellular metabolism regulating protein synthesis.

Firstly, we hypothesized that reduced cellular energy status by glucose starvation leads to inactivation of p70S6K. It has been reported that reduced intracellular ATP/ADP ratios stimulate AMP‐activated protein kinase (AMPK) and hence inhibit the mTORC1/p70S6K pathway. However, in our study, the concentration of intracellular ATP neither decreased by glucose starvation nor increased by glucose re‐addition. These results suggest that the intracellular concentration of ATP is not associated with the phosphorylation levels of p70S6K during glucose starvation or in response to glucose re‐addition.

Glucose deprivation is a known factor for stimulating protein breakdown. The autophagy‐lysosome system [9, 10, 11] and ubiquitin‐proteasome system [28, 29] are also reported to be accelerated by glucose starvation. Thus, we examined the role of the autophagy‐lysosome system and the ubiquitin‐proteasome system in our glucose re‐addition‐induced activation of p70S6K by using pharmacologic inhibitors. We treated the cells with bafilomycin and confirmed the disruption of lysosomal activity by evaluating the increasing ratio of LC3‐II/LC3‐I [26, 27]. Bafilomycin‐mediated inhibition of autophagy also attenuated the phosphorylation of p70S6K in response to glucose re‐addition. On the other hand, inhibition of proteasome activity by MG132 did not affect the phosphorylation of p70S6K by glucose re‐addition, but resulted in accumulation of multi‐ubiquitinated proteins. These results suggest that autophagy under glucose starvation enhances the signaling of protein translation initiation in response to glucose re‐addition in C2C12 myotubes and that the ubiquitin‐proteasome system is not involved in this process.

The effects of autophagy inhibition on mTORC1 signaling under amino acid deprived conditions in C2C12 myotubes have been reported; results showed that autophagy is required to sustain mTORC1 signaling during amino acid starvation [30]. In the present study, glucose starvation for 24 h with autophagy inhibition further diminished the phosphorylation levels of p70S6K, in line with the previous report. It is known that lysosomes facilitate mTOR activation by generating an internal pool of amino acids through protein degradation [31]. In the present study, adequate amounts of amino acids were presented in culture medium even during glucose starvation and the basal level of phosphorylation of p70S6K was significantly reduced by inhibiting autophagy. Thus, further study is required to determine the effect of intracellular concentrations of amino acids during glucose starvation.

Although the phosphorylation levels of p70S6K at threonine 389 is reported to be correlated with the protein translation initiation [25], the actual protein synthesis rate should be confirmed using labeled amino acids or the SUnSET assay [32]. Moreover, the effect of repeated glucose starvation and restoration on myotubes should be explored in future.

In the present study, we assessed the effect of glucose restoration following 24‐h nutrient stress by glucose starvation alone. Further studies are required to evaluate the optimum starvation period to maximize the effect of glucose restoration on protein synthesis. We believe that adequate nutritional stress (such as intermittent fasting) might be useful for improving skeletal muscle mass and function.

## Conflict of interest

The authors declare no conflict of interest.

## Author contributions

NN, SK, and KH designed the study and wrote the manuscript. SK, NI, and SI performed the experiments and analyzed the data.

## Data Availability

The authors state that all data will be available from the corresponding author upon reasonable request.
